# Exploring the Competency of the Jordanian Intensive Care Nurses towards Endotracheal Tube and Oral Care Practices for Mechanically Ventilated Patients: An Observational Study

**DOI:** 10.5539/gjhs.v5n1p203

**Published:** 2012-12-24

**Authors:** Abdul-Monim Batiha, Ibrahim Bashaireh, Mohammed AlBashtawy, Sami Shennaq

**Affiliations:** 1 Faculty of Nursing, Philadelphia University, Jordan; 2 Princess Salma Faculty of Nursing, AL al-Bayt University, Jordan

**Keywords:** intensive care nurse, intubated patient, Jordan, oral care practice, observational study, oropharynx

## Abstract

Oral care is an important feature of nursing; it is known that oropharynx is considered the main reservoir of bacterial colonization, so the removal of oral infection is a major duty of all health care providers, particularly nurses. We performed this study to explore endotracheal tube and oral care practices for mechanically ventilated patients of Jordanian intensive care nurses, and to study Jordanian intensive care nurses’ practices during, prior to, and post endotracheal tube and oral care for mechanically ventilated patients. Endotracheal tube and oral care of Jordanian intensive care nurses for mechanically ventilated patients was compared with recommendations for endotracheal tube and oral care of American Association of Critical Care Nurses and guidelines of Centers for Disease Control and Prevention. Non- participant structured observational design was conducted using a 24 -item structured observational schedule. The findings show that nurses different in their oral care practices; did not follow American Association of Critical Care Nurses recommendations; and therefore delivered lower-quality oral care than predictable. Important inconsistencies were observed in the nurses’ hyperoxygenation, respiratory assessment techniques and infection control practices.

## 1. Introduction

Oral care is an important aspect of nursing, which has an impact on the health, comfort, and well-being of mechanically ventilated patients (MVPs). A professional knowledge and skills is required to perform Oral care for MVPs (Kathleen, 2002).

Oral care has a deep influence on the overall health. Colonization of the oropharyngeal is associated with numerous systemic diseases, including chronic obstructive pulmonary disease, endocarditis, and bacteremia (Stonecypher, 2010), cardiovascular disease (Munro, 2004; Li et al., 2000; Fowler et al., 2000), In the critical care units (CCUs), interest in relationship of oral care to systemic disease has concentrated on the development of ventilator-associated pneumonia (VAP) (Graves et al., 2010; Wip & Napolitano, 2009). VAP leads to extended critical care unite (CCU) stay, costs and increased mortality (Kearns et al, 2009).

The oropharynx is usually considered the main reservoir of bacterial colonization in the upper airways because oropharyngeal colonization by aerobic pathogens occurs very rapidly in ICU patients. Nurses usually prioritize care to immediate medical problems. Moreover, critical care nurses may be hesitant to deliver oral care to MVPs because endotracheal tube (ETT) may limit entree to the oral cavity. The opportunity of removing or moving an ETT is also a concern (Stiefel et al., 2000).

Oral care is frequently considered chiefly an intervention for clients’ comfort (Yeung & Chui., 2010), a characteristic that may decrease its frequency and priority. Knowledge about the type and frequency of oral care provided to MVPs will improve nursing interventions that may increase positive outcomes in these patients (Grap et al., 2003). The removal of infection is a major duty of all health care providers, particularly nurses, as they have the most frontline contact with the patient. It is essential to teach nurses the scientific care of the teeth and proper oral care to keep oral mucosa moist, clean, and free from secretions that can lead to infection. Thus, avoiding deterioration of oral health is a significant part of nursing care of acutely ill clients (Kathleen, 2002). Furthermore, nursing practice continues to be provided based on habitual practice, tradition and individual nurses’ preferences (Kathleen, 2002).

## 2. Methods

### 2.1 Study Objectives

Exploring the competency of the Jordanian intensive care nurses towards ETT and oral care practices for MVPs.

To compare ETT and oral care of Jordanian intensive care nurses for MVPs with recommendations for endotracheal tube and oral care of American Association of Critical Care Nurses (Scott & Vollman, 2011) and guidelines of Centers for Disease Control and Prevention (CDC) (CDC, 2004).

### 2.2 Hypothesis

Jordanian intensive care nurses do not follow guidelines for ETT and oral care as recommended by Scott & Vollman (2011) and CDC recommendations (CDC, 2004).

### 2.3 Design

To achieve the above objectives, non-participant structured observational design was used, in which the researcher is a non-participant observer who records the phenomena under examination using a framework for data collection. This framework was developed prior to commencing the study. The researchers aim is to devise a tool that will facilitate the systemic collection of data in a way that will, as far as possible, limit the subjectivity of the observer, therapy enhancing validity and reliability.

### 2.4 Setting

This study was conducted in ten intensive care units in ten major hospitals in Jordan: 3 private hospitals, 2 university teaching hospitals, 2 military hospital and 3 public sector hospitals (Ministry of Health). This is a representative cross-section of the health sector and intensive care nurses in Jordan. This study took place between September 2010 to April 2011.

### 2.5 Sample

The sample includes 150 intensive care nurses who perform ETT and oral care practice for MVPs. The sampling unit was the ETT and oral care event itself. Event sampling which involves the selection of integral behaviors or events was hypothetical the best suitable technique of observation for the changeable nature of the ETT and oral care procedure (Polit, 2006). Using quota sampling, a total of 150 individual ETT and oral care events were observed, whereby each nurse performed only one event. During Quota sampling the investigator can guide the selection of subjects so that the sample contains an suitable number of cases from each stratum (Laura et al., 2010), the strata in this study are ICUs nurses from 3 private hospitals, 2 university teaching hospitals, 2 military hospital and 3 public sector hospitals (Ministry of Health). In this study, event (ETT and oral care practice) sampling was used and the sequenced nature of ETT and oral care practice was followed. In addition, “event sampling requires researchers to either have knowledge about the occurrence of events or be in a position to wait for or precipitate their occurrence” (Laura et al., 2010). Exclusion and inclusion criteria were preserved.

#### 2.5.1 Inclusion Criteria

Intensive care nurses (registered or associate degree nurses) with at least one year of ICU experience were included in this study; this experience was required because if nurses have less than one year’s experience it is anticipated that they do not possess the knowledge or skills required for the procedure observed.

### 2.6 Data Collection

By 24 item structured observational schedule, data were collected which was adapted from (Scott & Vollman, 2011) and CDC guidelines (CDC, 2004) (Appendix). Pilot study was done to identify any problems that might possibly affect the research process and practically of the observational schedule. No modifications were made to the instrument based on the pilot study. The research assistants (observers) are registered nurses who received systematic training and education prior to data collection to follow the ETT and oral care according to Scott & Vollman (2011) and CDC recommendations (CDC, 2004).

To prevent Hawthorne effect, the research assistants deliberately planned the observations throughout eight months of the observational study. Observations were done during A shift (7am-3pm), B shift (3pm-11pm) and C shift (11pm-7am).

All days, shifts and ICU sites had equivalent chances of being selected for each time. Before access into the intensive care units (ICUs), each ICU director was knowledgeable as to why, when they would be ICU, but ICU staffs were not informed as to when the researchers would be present in the unit.

### 2.7 Validity and Reliability

Observation check list was sent for appraisal to university doctors’ tutors in critical care nursing, arrange of experts in critical care setting and experts in nursing research to assess the validity. Following observers’ training a pilot study was carried out to test the inter-rater reliability by the use of a second observer, and no significant differences were identified.

### 2.8 Ethical Consideration

The scientific research committee at Philadelphia University approved the methodology and provided financial support to carry out this research. Access to the field was obtained from the institution review boards in the hospitals included in this study. Written informed consent was obtained from each participant nurse before the data collection process. They were assured that confidentiality, anonymity, and the right to withdraw from the study would be honored. In addition, voluntary participation was ensured. The purpose of the study was explained and feedback about study results will be given to all participants.

### 2.9 Statistical Analysis

Data collected were coded and tabulated according to Statistical Package for the Social Scientists (SPSS, version 17) software. Descriptive statistics included percentages for nominal-level data and frequency ratings. To exam the null hypothesis and compare ETT and oral care of Jordanian intensive care nurses for MVPs with recommendations guidelines a one-sample t-test was used.

### 2.10 Quality of Treatment

The following assessment scale was used: a score of 22 (the highest possible score) shows that the participant practice of endotracheal and oral care matched the standards set by the Scott & Vollman (2011) variable, representing “recommended ETT and oral care practice”. Two of the original 24 scale items were not applicable to our patients because none of them were nasally intubated. Every 24 items on the schedule was weighted with 0 and 1 (zero=not done and 1=done). This was elaborated by computing the summation of the maximum possible scores for each observation, which was established as being 22. The number 22 therefore represented perfect adherence to recommended ETT and oral care (Scott & Vollman, 2011) and CDC recommendations (CDC, 2004). The higher the intensive care nurse scored based on the observation check list, the closer the participants matched the recommended practices. Likewise, the lower the intensive care nurse scored, least expected adherence to recommended practices. This score represented the “quality of treatment”. For the purposes of the data analysis, the score was divided into four parts: practices prior to oral care, during and after the oral care procedure as well as patients’ monitoring and care.

## 3. Results

The result were divided into 5 main elements according to the observational schedule, including practices prior to oral care, oral care event, post oral care and finally patient monitoring and care.

### 3.1 Practices Prior to Oral Care

In relation to practices prior to oral care, the results identified that only 40 nurses (27%) wash their hands (Table 1). In relation to wearing gloves before oral care, the vast majority (N=147, 98.0%) of ICU nurses wore gloves. In addition, over three-quarters of the participants were observed to hyperoxygenate (N=122, 81.3%) before suctioning. It was noted that the vast majority of them (N=142, 94.7%) conducted endotracheal suctioning if suctioning was clinically indicated. Furthermore, participants loosened or removed tapes (N=124, 82.7%) prior to oral care. Finally, over half of the sample (N=85, 56.7%) were observed to remove bite-block or oropharyngeal airways before administering oral care, which is used to prevent biting down on the ETT and occluding airflow. (N=37, 24.6%) of patients had no oropharyngeal airway applied at all.

**Table 1:** Practices prior to oral care

Variable	(n=150)
Hands are washed	
Yes	40(27%)
No	110(73%)
Gloves wearing	
Yes	147(98.0%)
No	3(2.0%)
Hyperoxygenation/hyperinflation	
Yes	122 (81.3%)
No	28 (18.7%)
Suction endotracheal tube	
Yes	142 (94.7%)
No	8(5.3%)
Old tape and ties are loosened and removed	
Yes	124(82.7%)
No	26(17.3%)
Oropharyngyngeal airway are removed	
Yes	85 (56.7%)
No	28(18.7%)
No oropharyngeal airway applied	37(24.6%)

n = sample number

### 3.2 The Oral Care Event

Regarding toothbrush for orally intubated patients (Table 2), no participants administer tooth brushing, and over one-third of them (N=60, 40%) did oral swap with hydrogen peroxide solution (H_2_O_2_) or 2% Chlorhexidine solution to clean the mouth every 2-4 hours for their patients.

**Table 2:** The oral care event

Variable	(n=150)
Toothbrush at least twice a day	
Yes	0(0.0%)
No	150(100.0%)
Oral swab with 1.5% H_2_O_2_ or 2% Chlorhexidine Solution q 2 to 4 hs.	
Yes	60(40.0%)
No	90(60.0%)

H_2_O_2_ = hydrogen peroxide; n = sample number

### 3.3 Post-Oral Care Practices

Post oral care practices results identified that the majority of participants (N=136, 90.7%) of the observed nurses complied fully with recommendations of oral care (Scott & Vollman, 2011) and CDC guidelines (CDC, 2004) in relation to frequent suction of the oral cavity/pharynx (Table 3). In addition, the oral tubes were moved to the other side by almost two- thirds of the participants (N=97, 64.7%), and the oropharyngeal airways were replaced along ETT by over half of participants (N=86, 57.3%); and reconfirming tube placement and noting position of tube at teeth or naris (N=124, 82.7%), and securing the position of the ETT was done by 86.7% (N=130). Finally, washing hands after finishing this procedure was performed by the vast majority of participants (N=144, 96.0%).

**Table 3:** Post-oral care practices

Variable	(n=150)
Suction oral cavity/pharynx	
Yes	136(90.7%)
No	14(9.3%)
Oral tube was moved to the other side of the mouth	
Yes	97(64.7%)
No	53(35.3%)
Oropharyngeal airway was replaced along ETT	
Yes	86(57.3%)
No	33(22.0%)
No oropharyngeal airway applied	31(20.7%)
The nurse ensured proper tube cuff inflation	
Yes	49(32.7%)
No	101(67.3%)
Reconfirm tube placement	
Yes	124(82.7%)
No	26(17.3%)
Secure the endotracheal tube in place	
Yes	130(86.7%)
No	20(13.3%)
Applying a mouth moisturizer	
Yes	117(78.0%)
No	33(22%)
Hand washing	
Yes	144(96.0%)
No	6(4.0%)

ETT = endotracheal tube; n = sample number

### 3.4 Patient Monitoring and Care

Patient monitoring and care identified that only 24 nurses (16%) didn’t elevate the head of the bed to 30^o^ when administering oral care (Table 4). The results also revealed that the majority of nurses (N=140, 93.3%) did ETT suctioning if clinically indicated, and the majority of the participants (N=123, 82%) observed the amount, type and color of secretions. Furthermore, the assessment of the oral cavity and lips, and the build-up of plaque was conducted by over three-quarters of the sample (n=119, 79.3%). Lastly, majority of participants reconfirmed tube placement, and noted the position of the tube at teeth or naris (n=134, 89.9%).

**Table 4:** Patient monitoring and care

Variable	(n=150)
Head of the bed elevated 30°	
Yes	126(84.0%)
No	24(16.0%)
Suctioning ETT if clinically indicated	
Yes	140(93.3%)
No	10(6.7%)
Monitored amount, type and color of secretions	
Yes	123(82.0%)
No	27(18.0%)
Oral cavity and lips assessed at least q 8 hs, and perform oral care q 2 to 4 hours and as needed	
Yes	118(78.7%)
No	32(21.3%)
Assessed buildup of plaque on teeth	
Yes	119(79.3%)
No	31(20.7%)
Reconfirm tube placement	
Yes	134(89.9%)
No	15(10.1%)

ETT, endotracheal tube; n = sample number

### 3.5 Quality of Treatment

The average of mean treatment quality for all participants was 16.49 (Table 5). The maximum average score was found in post-oral care practices (mean 5.89, SD 1. 417) and the lowest average of mean score were found in the oral care event measures (mean .46, SD .563).

**Table 5:** Quality of treatment

Variable	Practices prior to oral care	The oral care event	Post-oral care practices	Patient monitoring and care	Quality of treatment
N	150	150	150	150	150
Mean	5.07	.46	5.89	5.07	16.49
Median	5.00	.00	6.00	5.00	15
Mode	6	0	6	6	17
Std.Deviation	.887	.563	1.417	1.157	2.11
Range	4	2	6	5	12
Minimum	2	0	2	1	7
Maximum	6	2	8	6	30

### 3.6 Testing the Null Hypothesis

To compare participants’ oral care practices with recommendations for ETT and oral care (Scott & Vollman, 2011) and CDC guidelines (CDC, 2004), a one-sample t-test was implemented, in which comparison between treatment quality observed with the ideal treatment quality score (Table 6). The test recognized significant differences between the quality of treatment and its subscales and the perfect score (representing recommended best practice). In all categories, except for post-oral care practices, the quality of treatment observed was significantly lower than the quality of treatment required (P< 0.01). This indicates that our study’s sample group only partially adhered to recommendations for ETT and oral care (Scott & Vollman, 2011) and CDC guidelines (CDC, 2004), when performing oral care practice, hence the null hypothesis is rejected.

**Table 6:** Comparisons between current practices with recommendations for oral care in the 2011 AACN procedure manual for critical care

Variable	Maximum potential score	Mean (actual score)	Standard Deviation (SD)	T	Df	p
Quality of treatment	22	16.49	2.73	-24.61*	149	.000
Practices prior to oral care	6	5.07	.887	-12.88*	149	.000
The oral care event	2	.46	.563	-33.49*	149	.000
Post-oral care practices	8	5.89	1.417	-.980*	149	.329
Patient monitoring and care	6	5.07	1.157	-9.88*	149	.000

* P< 0.01

## 4. Discussion

No ideal standard exists for ETT and oral care for MVPs. We chose Scott & Vollman (2011) and CDC guidelines (CDC, 2004) as competency standards for ETT and oral care practices for MVPs because it considered as a comprehensive guidelines that based on evidence base practices and was used by other study (Laura et al., 2010).

A quasi-experimental research study was conducted in Jordan by Batiha (2010), to determine the effect of implementing an oral care protocol on the occurrence of VAP. He found that implementing comprehensive oral care protocol to improve oral health status have been shown to be effective in reducing the incidence of VAP, as evidenced by reducing the occurrence of VAP from 40% in the control group to 16.7% in the intervention group.

Recommendations for oral care (Scott & Vollman, 2011) and CDC guidelines (CDC, 2004) suggest toothbrushing at least twice a day, and oral swab with 1.5% H_2_O_2_ to clean the mouth every 2 to 4 hours. The result of this study in contrary of European study which they found 41% of the critical care nurse brushed the patients’ teeth (Rello et al., 2007), and other study in which toothbrushing was used in 50.8% of the nurses (Soh, 2012) comparing to no one of Jordanian critical care nurses brushed the patients’ teeth. This may be interpreted by toothbrushing is not part of routine oral care practices in Jordanian hospitals, and unavailability of supplies, equipment and lack of time all of these reasons affect the type and quality of oral care given by nurses (Curzio & McCowan, 2000; Moore, 1995).

Our findings are in-line with earlier studies that found there is commonly lack of toothbrushes and toothpaste in hospital setting (Binkley et al., 2004; Sole et al., 2003).

The fear of removing or dislocating the tube is also a concern. Providing oral care usually affected by wrong thinking that oral care has a lower priority and contributes less to patients’ health and well-being (Grap, 2009).

Time limit for oral care is considered as a significant factor. Shortage of ICUs nurses in Jordan, and consequent overburden, is an important factor in providing oral care which is in line with other studies (Aiken et al., 2002; Steinbrook, 2002). When nurses are busy and lack of time, oral care is the first procedure to be postponed (Wardh et al., 2000). Exploring the association between nurse nosocomial infection rates, overcrowding and staffing found that nurse staffing is considered as an important effects on patient outcomes (Katherason et al., 2009).

Despite a lot of evidence base practice on the adverse effect of suctioning- induced hypoxemia (Thompson, 2000; Day & Wilson-Barnett, 2002I), 28 (18.7%) nurses still not hyperoxygenation and suction endotracheal tube. Day et al. (2002II) described comparable results, whereby only 2 from 10 subjects in their study were observed to provide hyperinflation/hyperoxygenation in clinical practice. These results are significant because they have direct implications for patient safety and reflect poorly on an essential aspect of nursing. Nosocomial infections are most common complication to patients in general (Burke, 2003). Boyce & Pittet (2003) suggested that nurses usually not wash their hands because of limited time and busy work especially in highly work area such as ICU and because of understaffing or overcrowding. Three nurses in this study were not observed to worn personal protective equipment’s like gloves during oral care. This may be explained by that wearing glove when performing oral care contradicts the need for recurrent hand washing. However, the literature clearly suggests that gloves do not replace the need for hand washing (Pratt, 2001).

### 4.1 Clinical Relevance

- ICU nurses in this observational study were often not adhering to the recommendations for ETT and oral care according to Scott & Vollman (2011) and CDC recommendations (CDC, 2004) in their current practice and consequently need continuous education related to evidence base ETT and care protocol.
- Nurses are in need for clinical practice to improve knowledge, skills and proficiency of oral care particularly brushing teeth, suctioning, oral assessment, infection control practices and oxygenation.
- Collaborative interactions with dental hygienists improve knowledge and abilities related to oral care
- The use of standardize oral assessment improve patient care quality and assess nurses in providing efficient oral care.

### 4.2 Study Limitations

Observation, as with all methods, has limitations and ethical implications (Parahoo, 1997). Although observation is a vital method of data collection, both unstructured and structured observations are vulnerable to biases. Human perceptual errors and inadequacies are a continuous threat to the quality of obtained information (Garrouste-Ortegas, 1997). Observation and interpretation are demanding tasks, requiring attention, sensation and perception, representing in the current study careful construction and pretesting of checklists and rating scales and the proper training and preparation of observers, all of which techniques play an important role in minimizing biases.

Hawthorne effect is considered as one of the main problems in this study, which means the influence of the observer on the observed, this may lead to change some participants their behavior in the presence of the observer. In this study, the participants’ practice may be of a lesser quality than suggested by the results of our findings. Also there were many aspects of oral care procedure that could not be assessed. It was impossible to know nurses’ reasons for their practices, such as oral cavity and lips being assessed for the buildup of plaque on teeth; it was not possible to identify what nurses assessed and how it was interpreted (Ames, 2011). This may have resulted in a wrong interpretation of the results.

## 5. Conclusions

Nurses did not implement the latest evidence as reported by recommendations for ETT and oral care (Scott & Vollman, 2011) and CDC recommendations (CDC, 2004) in their current practice. Therefore intensive care nurses should take educational intervention regarding ETT and oral care and use standardized oral care protocols to decrease VAP incidence rate on their units. The results of this study raised interesting questions on current oral care practices of intensive care nurses.

## Appendix

Observational checklist

**Part I: Practices Prior to Oral Care**

1. Hands are washed.
  A. Yes B. No
2. Personal protective equipment’s (e.g. Gloves) are worn.
  A. Yes B. No
3. Hyperoxygenation/hyperinflation prior to suctioning
  A. Yes B. No
4. Suction endotracheal tube.
  A. Yes B. No
5. Old tape and ties are loosened and removed.
  A. Yes B. No
6. If patient is nasally intubated, the nurse cleaned around endotracheal tube using saline-soaked gauze or cotton swabs.
  A. Yes B. No
7. If patient is orally intubated oropharyngeal airway or mouth Gag (acting as bite-block) are removed.
  A. Yes B. No

**Part II: The Oral Care Event**

8. Perform oral care using Pediatric toothbrush or Adult (soft) toothbrush at least twice a day. Gently brush patient’s teeth to clean and remove plaque from teeth.
  A. Yes B. No
9. Oral swab with 1.5% Hydrogen peroxide solution to clean mouth every 2 to 4 hours.
  A. Yes B. No

**Part III: Post-Oral Care Practices**

10. Suction oral cavity/pharynx.
  A. Yes B. No
11. Oral tube was moved to the other side of the mouth.
  A. Yes B. No
12. Oropharyngeal airway (bit-block) was replaced along the endotracheal tube (*to prevent biting, minimize pressure areas on lips, tongue, and oral cavity)*.
  A. Yes B. No
13. The nurse ensured proper tube cuff inflation using minimal leak volume or minimal occlusion volume.
  A. Yes B. No
14. After oral care, the nurse reconfirm tube placement, and note position of tube at teeth or naris *(common tube placement at teeth is 21 cm for women and 23 cm for men)*.
  A. Yes B. No
15. After oral care, the nurse secure the endotracheal tube in place (according to institutional standard) *(to prevent inadvertent dislodgment of the tube)*
  A. Yes B. No
16. With each cleansing, applying a mouth moisturizer to the oral mucosa and lips to keep tissue moist?
  A. Yes B. No
17. Hands are washed.
  A. Yes B. No

**Part IV: Patient Monitoring and Care**

18. Head of the bed elevated at least 30 degree unless contraindicated.
  A. Yes B. No
19. Suctioning endotracheal tube if clinically indicated.
  A. Yes B. No
20. The nurse monitored the amount, type and color of secretions.
  A. Yes B. No
21. If patient is nasally intubated, the nurse monitor for nasal drainage.
  A. Yes B. No
22. The nurse assesses the oral cavity and lips at least every 8 hours.
  A. Yes B. No
23. During oral care, the nurse assesses buildup of plaque on teeth or potential infection related to oral abscess?
  A. Yes B. No
24. The nurse reconfirm tube placement, and note position of tube at teeth or naris. Retape or secure endotracheal tube at least once per day for soiled or loose securing devices.
  A. Yes B. No
